# Supplementary material for: Apalutamide Monotherapy in Metastatic Hormone-Sensitive Prostate Cancer: A Viable Alternative to First-Generation Anti-Androgen Agents to Avoid the Flare Phenomenon and an Effective Treatment for Achieving Early PSA Response
Source: Cancers (Basel). 2025 Aug 5;17(15):2573. doi: 10.3390/cancers17152573 (PMC12346201; doi:10.3390/cancers17152573)
Supplement: Supplementary file 1 [file cancers-17-02573-s001.zip › cancers-3728570-supplementary.pdf]

Supplementary Materials

Table S1. Testosterone levels and proportion of patients achieving testosterone reduction at 14 and 28 days, overall and according to disease volume.

	All patients (n=27)	Low volume (n=17)	High volume (n=10)	p-value b
Testosterone at baseline, Mean (SD)	6.56 (4.46)	6.62 (4.63)	6.46 (4.40)	0.94
Testosterone at day 14, Mean (SD)	6.58 (4.42)	7.26 (4.69)	5.41 (3.86)	0.33
Testosterone at day 28, Mean (SD)	2.40 (3.38)	2.75 (3.51)	1.80 (3.23)	0.63
**Any testosterone decreases at day 14** [a]	n (%)			
No	21 (77.8)	13 (76.5)	8 (80.0)	
Yes	6 (22.2)	4 (23.5)	2 (20.0)	0.99
**Achieved a >50% testosterone decrease at day 14** [a]				
No	26 (96.3)	17 (100.0)	9 (90.0)	
Yes	1 (3.7)	0 (0.0)	1 (10.0)	0.37
**Achieved a >90% testosterone decrease at day 14** [a]				
No	26 (96.3)	17 (100.0)	9 (90.0)	
Yes	1 (3.7)	0 (0.0)	1 (10.0)	0.37
**Any testosterone decreases at day 28** [a]				
No	3 (11.1)	3 (17.6)	0 (0.0)	
Yes	24 (88.9)	14 (82.4)	10 (100.0)	0.27
**Achieved a >50% testosterone decrease at day 28** [a]				
No	7 (25.9)	6 (35.3)	1 (10.0)	
Yes	20 (74.1)	11 (64.7)	9 (90.0)	0.20
**Achieved a >90% testosterone decrease at day 28** [a]				
No	19 (70.4)	12 (70.6)	7 (70.0)	
Yes	8 (29.6)	5 (29.4)	3 (30.0)	0.99

[a] As compared to day 0. [b] p-value for comparison between low and high volume disease. SD: standard deviation.

Table S2. Adverse events reported during treatment in 27 patients.

	All patients (n=27) [a]	Grade [b]		
**Event**	**n (%)**	**G1**	**G2**	**G3**
Any adverse event	16 (59.3)	5	4	7
Hypertension	3 (11.1)	2	0	1
Cardiopathy	1 (3.7)	NR	NR	NR
Diabetes	0	-	-	-
Neurological Events	0	-	-	-
Respiratory Events	0	-	-	-
Cutaneous Events	11 (40.7)	3	3	5
Hypothyroidism	1 (3.7)	NR	NR	NR
Other adverse events	2 (7.4)	0	1	1

NR: not reported. [a] Two patients reported two different toxicities, for a total of 18 events occurring in 16 patients. [b] For two toxicity events, the corresponding grade was not reported.
